# The impact of ambulatory care spending, continuity and processes of care on ambulatory care sensitive hospitalizations

**DOI:** 10.1007/s10198-022-01428-y

**Published:** 2022-01-29

**Authors:** Wiebke Schuettig, Leonie Sundmacher

**Affiliations:** Chair of Health Economics, Technical University of Munich, Georg-Brauchle-Ring 60/62, 80992 Munich, Germany

**Keywords:** Germany, Ambulatory care, Health care costs, Hospitalizations, Continuity of care

## Abstract

Ambulatory care sensitive hospitalizations are widely considered as important measures of access to as well as quality and performance of primary care. In our study, we investigate the impact of spending, process quality and continuity of care in the ambulatory care sector on ambulatory care sensitive hospitalizations in patients with type 2 diabetes. We used observational data from Germany’s major association of insurance companies from 2012 to 2014 with 55,924 patients, as well as data from additional sources. We conducted negative binomial regression analyses with random effects at the district level. To control for potential endogeneity of spending and physician density in the ambulatory care sector, we used an instrumental variable approach. We controlled for a wide range of covariates, such as age, sex, and comorbidities. The results of our analysis suggest that spending in the ambulatory care sector has weak negative effects on ambulatory care sensitive hospitalizations. We also found that continuity of care was negatively associated with hospital admissions. Patients with type 2 diabetes are at increased risk of hospitalization resulting from ambulatory care sensitive conditions. Our study provides some evidence that increased spending and improved continuity of care while controlling for process quality in the ambulatory care sector may be effective ways to reduce the rate of potentially avoidable hospitalizations among patients with type 2 diabetes.

**Supplementary Information:**

The online version contains supplementary material available at 10.1007/s10198-022-01428-y.

## Introduction

A large proportion of today’s hospital admissions are thought to be avoidable through effective management and timely treatment in the ambulatory care sector. So-called ambulatory care sensitive conditions (ACSC) have become a measure to assess access, quality and performance of care, as an indicator of quality in ambulatory care [1, 2] and are sometimes used as a rough estimate of the proportion of potentially avoidable hospitalizations in health care systems. These hospital admissions are not only undesirable for patients but also disruptive and costly for payers and purchasers of care, such as statutory or private health insurers, or clinical commissioning groups.

Ways to reduce these potentially avoidable hospitalizations include controlling acute episodes of an illness, managing a chronic condition effectively, or preventing a disease in the first place [3]. Most of these approaches involve an increase in the use of ambulatory care resources. In general, it has been assumed that increasing the volume of health services provided, for example through more ambulatory care spending, will lead to improvements in the health status of a population [4]. This raises the question of whether an increase in ambulatory care spending can reduce hospitalizations for ACSC.

Considering the budget constraints in most health care systems, several empirical studies have explored the relationship between health care resourcing and patient outcomes with the aim of estimating the extent to which an increase in the volume of health care services yields benefits for patients [5].

A number of studies have examined the relationship between the use of ambulatory care resource and hospitalizations while specifically focusing on ACSC. Many of these have reported a negative relationship between physician density and ambulatory care sensitive hospitalizations (ACSH) [6–10]. A systematic review of studies examining the effects of primary health care resourcing on diabetes-related hospitalizations concluded that access to ambulatory care was negatively associated with hospitalizations, whereas evidence of an association between the *use of* ambulatory care and hospitalizations was inconclusive [11]. The authors recommended that future studies adjust adequately for patients’ health status and disease severity. Sundmacher and Kopetsch [12], in turn, used aggregate-level data to investigate the impact of the volume of office-based care on hospitalizations for ACSC in Germany. They found that increasing the volume of medical services reduced the rate of hospitalizations, albeit with diminishing marginal returns.

This study focuses on patients with type 2 diabetes. The chronic disease is an ACSC and one of the top ten causes of death worldwide. Its prevalence has quadrupled within the past 36 years and is likely to increase further [13]. Diabetes is also a major cause of morbidity and is associated with more and longer hospital stays and an increased number of surgical complications [14–16]. In addition, diabetes complicates the diagnosis and treatment of other medical disorders [17]. Ambulatory care plays a key role in the management of patients with diabetes [18]. This includes monitoring the disease, taking preventive measures, administering medical treatment, and giving lifestyle advice [19]. Good continuity of care and the adherence to clinical practice guidelines are well-documented to be associated with fewer hospitalizations among patients with diabetes [20, 21]. Coordination and continuity of care have the potential to reduce the duplication of services and overall health care costs, and to improve health outcomes [22, 23]. The results of earlier studies suggest that improved continuity of care can result in better control of chronic diseases, higher patient satisfaction, and fewer emergency department visits and hospitalizations [24–27]. Especially in systems of statutory health insurance, such as that in Germany, where no gatekeeping is in place, care coordination becomes even more relevant [28]. Measures to improve the quality of care may be complicated or hampered by fragmentation of the ambulatory sector. Moreover, incentives to contain costs can be located within individual physicians.

Specifically in the German health care system, ambulatory care is characterized by professional autonomy and a large proportion of office-based physicians in solo practices [29]. In the statutory health system, no gatekeeping is in place, and patients can seek ambulatory care at any GP or specialist office-based physician at any point in time. Coordination of care for chronically ill, multimorbid patients is a major challenge in the health care system [30]. Problems in coordination of care can lead to increased ambulatory care costs and negative consequences for patients, such as redundant, diverging procedures and deterioration in health states [31, 32].

Earlier studies have investigated the effects of ambulatory care resourcing on ambulatory care sensitive hospitalizations using aggregated data or have analyzed the effects on health outcomes, of processes of care for specific diseases. Our study contributes to the literature by investigating the effect of ambulatory care spending on hospitalizations among patients with type 2 diabetes using measures of care continuity and process indicators at the level of individual patients. In doing so, we adjust for a number of known risk factors for hospitalizations among this patient group.

## Methods

### Study setting and data sources

This is an observational study based on insurance claims data from 2012 through 2014 provided by the scientific research institute of the regional statutory health insurers (AOKen) in Germany, which cover approximately 36% of the population insured within the country’s system of statutory health insurance [33]. The data used contain information about inpatient and outpatient visits and spending, prescriptions, and diagnoses among a sample of patients older than 18 years and with at least two confirmed diabetes diagnoses in two different quarters. This sample was drawn from a larger sample of 3.08 million patients with a diabetes diagnosis. Our sample was smaller, because data protection regulations did not allow the statutory insurers to provide us with the full data set. In addition, to ensure a complete history of patients’ health care, we excluded patients who had died during or were not insured throughout the entire period 2012–2014.

We excluded patients who were living with HIV, had a diagnosis of metastatic cancer, were on dialysis, were dependent on opioids, or were receiving intensive nursing or palliative care because of the specifics of these treatments/diseases and the high likelihood of hospitalization independent of the ambulatory care treatment as well as different treatment goals. In addition, patients with diseases whose treatment is considered to be very expensive were excluded from further analyses (i.e., high-risk patients). In identifying high-risk patients, we concentrated on the diagnoses of the high-risk patient pool in former versions of the morbidity-based risk adjustment scheme in Germany [34, 35]. We report results including the patients with HIV, metastatic cancer, dependent on opioids, and high-risk patients in sensitivity analyses later in the appendix. We assumed that a hospitalization would be most likely for these patients and that presumably timely and effective ambulatory care was not able to prevent these hospital cases.

**Fig. 1:**
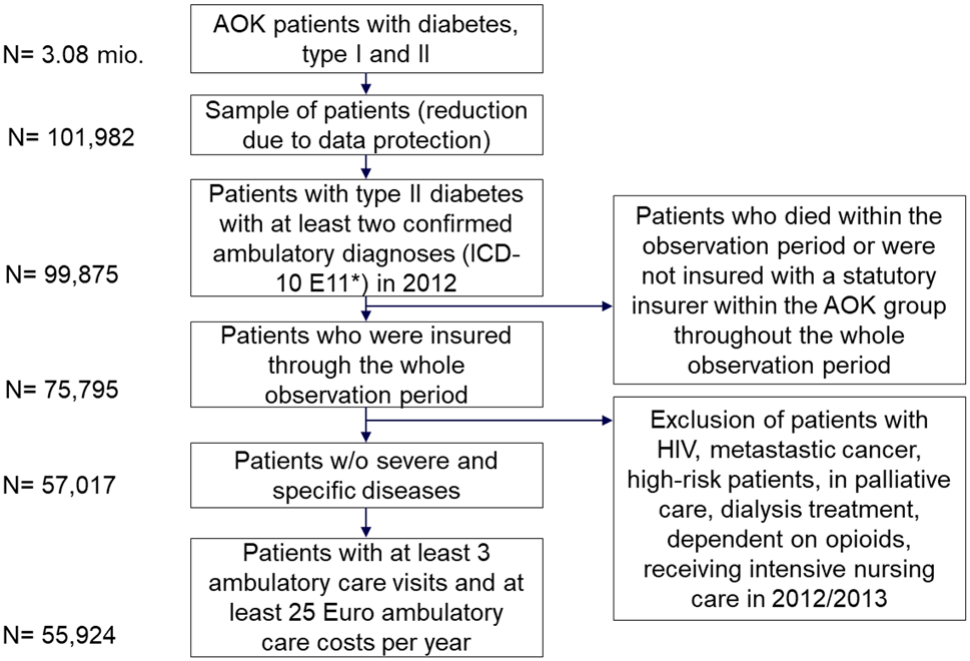
Data selection for the analysis

We expected that treatments received over the course of 2 years would impact upon health outcomes in the following year. Therefore, we defined an observation period (2012–2013) and a period for potential hospitalizations (2014). To compute valid continuity scores and due to plausibility considerations, we excluded patients who had made less than three ambulatory care visits to GPs during the observation period of 2 years. Owing to plausibility considerations, we also excluded patients for whom the statutory health insurers spent less than 25 Euro on ambulatory care per year during the observation period, because three visits to ambulatory care would already exceed that value. Figure 1 illustrates the process of selecting data for the analysis.

In addition, we used district-level data from the Federal Institute for Research on Building, Urban Affairs and Spatial Development [36] (physician density, unemployment rate and hospital density) and the Science Council [37] (medical students in a region) in our analyses to inform on structural variables, such as the physician density.

### Dependent variable

Our dependent variable was the number of hospitalizations due to ACSC. Patients with type 2 diabetes are at high risk of developing complications and comorbidities as the disease progresses [38], and several studies have shown that type 2 diabetes patients suffer from both a greatly increased all-cause hospitalization rate and diabetes-related hospitalization rate [39–41]. Complications and hospitalizations of individuals with diabetes can potentially be avoided and delayed through ambulatory care [38], which includes the prevention of diseases, controlling acute episodes of illnesses, and managing chronic conditions effectively.

To account for the fact that patients with type 2 diabetes experience a higher risk of vascular complications and morbidity related to increased need for ambulatory care [42], we used a list of hospitalizations following ACSC as dependent variable in our analysis. The range of hospitalizations is thought to capture all potentially avoidable hospitalizations that might result from a deterioration in health caused by a health state that could potentially have been avoided with ambulatory care. This list comprised 22 conditions specific to the German health care sector (see Table 2), resulting from potentially inadequate ambulatory care treatment [44].

We defined a hospitalization as a hospital admission that lasted at least 1 night. A hospitalization was defined as ambulatory care sensitive if the main diagnosis for the hospital admission in 2014 was coded as one of the included diagnosis groups (see Table 1). In addition, diabetes had to be at least a secondary diagnosis for the hospital admission so that we could be certain that patients were also treated because of diabetes.

**Table 1:** Summary statistics

Variable	Main sample (*N* = 55,941)	Sample including high-risk patients (*N* = 56,881)
	Mean (SD)	Mean (SD)
Ambulatory care sensitive hospitalizations	0.131 (0.453)	0.128 (0.450)
Ambulatory care spending	1641.08 (1028.64)	1657.11 (1052.99)
COCI (GP, internists)	0.714 (0.249)	0.714 (0.249)
COCI (GP)	0.743 (0.247)	0.742 (0.247)
COCI (GP, internists, eye specialist, diabetic specialist, specialist for nephrology)	0.6076 (0.237)	0.6046 (0.238)
MMCOCI	0.9429 (0.067)	0.9426 (0.068)
Visits (GPs, internists)	30.71 (16.47)	30.81 (17.25)
Visits (GPs)	29.70 (16.04)	29.77 (16.81)
Visits (GPs, internists, eye specialists, diabetic specialists, specialists for nephrology)	33.87 (17.66)	34.09 (18.43)
Age	66.46 (11.03)	66.50 (11.03)
Process indicator	0.048 (0.213)	0.048 (0.214)
Gender (= men)	51%	51%
ATC agents	1.76 (0.62)	1.76 (0.62)
Multimorbidity	6.9628 (3.351)	7.7176 (3.393)
DSI	3.37 (2.37)	3.39 (2.37)
Hypertension	92.11%	92.11%
Hazardous alcohol consumption or smoking	14.78%	14.81%
Depression	28.04%	28.13%
Obesity	47.51%	47.50%
Cancer	15.57%	16.60%
Sleeping disorder	19.83%	19.92%
Prior hospitalizations	0.404 (0.863)	0.407 (0.868)
Insulin prescription	23.47%	23.55%
Unemployment rate per district	7.18 (3.29)	6.95 (2.97)
Internist density per district	23.23 (8.29)	23.47 (8.31)
Spending per district	786.09 (89.22)	786.28 (89.21)
Medical students per district	10.59 (31.95)	10.64 (32.09)
Hospital bed density per district	61.88 (30.25)	60.99 (29.56)

### Ambulatory care spending, continuity of care and process indicators

Our primary explanatory variable was total ambulatory care spending per patient. We decided to include all ambulatory spending for a patient for two reasons: First, we assumed that diabetes would affect any treatment in ambulatory care. Therefore, the inclusion of all ambulatory care spending was a way to ensure a patient-oriented approach to our analysis. Second, ambulatory care spending is billed to statutory health insurers primarily in the form of quarterly capitation-type lump sums in Germany [28, 45], meaning that the incremental spending on the treatment of one disease cannot be measured without making crude assumptions. Spending was defined as cost to the statutory health insurer, which can be seen as an indicator for sector-specific resource use for the ambulatory care treatment of diabetes patients from the payer perspective. Patient co-payments, prescriptions, and private payments are, therefore, not included in our analyses. Out-of-pocket spending, however, is comparably low in Germany [46]. We hypothesized that, in case the care received was effective, hospitalizations would decrease as ambulatory care spending increases.

We aimed to depict continuity of care in our analyses, because measures to reduce ACSH at the systems level include interventions to improve the continuity of care [21, 47]. We assumed based on prior empirical literature that continuous care would decrease the risk of a hospitalization [25–27].

In our study, we operationalized continuity of care using the continuity of care index of Bice and Boxerman 1977. It measures the dispersion of visits and quantifies the percentage of visits to distinct providers. The index is commonly used [49, 50] and reflects both the total number of visits and the number of health care providers the patients sees [48]. It was used, because we hypnotized that continuity of care with the relevant providers would have positive effects on the treatment of patients in a system without gatekeeping and a lack of coordination. The index is calculated by dividing the sum of the squared number of visits per provider minus the total number of visits to all providers during the observation period by the number of all visits minus the number of all visits minus one.

Continuity of Care Index (COCI) =$\frac{{\mathop \sum \nolimits_{j = 1}^{k} n^{2} - N}}{{N\left( {N - 1} \right)}}$.

(*k* = number of providers, *n* = number of visits to provider; *N* = total number of visits to all providers).

The index ranges from 0 to 1 and increases when more visits are made to a smaller number of providers. In this study, we considered only those visits in 2012/2013 that were made to diabetes-relevant physicians within primary care (i.e., GPs and internal medicine physicians/internists). Alternative measures of continuity of care (COCI with GPs only, COCI with a wider set of physicians and the modified continuity index) are applied in the supplementary material. In addition, we controlled for the number of visits to these physicians based on the number of days for which physicians billed a service.

We also operationalized indicators reflecting processes of care within the observation period. Individuals with diabetes require systematic and interdisciplinary regular care [18]. We selected a composite process indicator for ambulatory care treatment based on recommendations from clinical practice guidelines, measurable using the routine data, and relevant to all diabetes patients. We indicated whether a patient received a yearly blood glucose test, a yearly microalbuminuria test, and at least one funduscopy examination within the observation period. These indicators are suggested for quality measurement in health care systems as they are reliably measureable and highly relevant for health outcomes of patients [51]. The gold standard for measuring control of blood sugar in diabetes is measuring glycated hemoglobin (hbA1c) [13]. A yearly hbA1c test and a test for microalbuminuria are recommended in medical guidelines and is seen as effective care for patients with diabetes [52–54]. The presence of microalbuminuria has been shown to predict progression to diabetic nephropathy, which is associated with fairly high mortality rates [54]. Earlier empirical studies that have examined the effect of processes of care on hospitalizations have, in particular, identified a negative association between these and regular hbA1c and microalbumin tests [18]. Furthermore, at least biennial eye screening is recommended for individuals with type 2 diabetes [53]. It allows for early detection and timely treatment of visual impairments [55]. To address aspects of disease prevention, we indicated whether patients received an influenza vaccination in each year of the observation period. Influenza vaccinations have been shown to reduce the risk of hospitalizations in type 2 diabetes patients by about 80% [56]. A yearly influenza vaccination in type 2 diabetes patients is recommended by the German permanent vaccination commission [57]. We hypothesized that patients who were treated according to medical guidelines and recommendations in the two observation years would have fewer hospitalizations. The composite measure was dichotomized and indicated when patients received all four recommended processes of ambulatory care. In sum, these process indicators represent a proxy for interdisciplinary, preventive, and comprehensive ambulatory care treatment following medical guidelines and recommendations.

### Control variables

Our study was informed by earlier research that identified adjustment for a number of known risk factors for hospitalizations among patients with type 2 diabetes [18, 58, 59].

We controlled for the age and gender of patients, as well as for disease severity and disease-specific risk factors. We determined whether patients were receiving insulin with at least one prescription each year during the observation period to control for disease severity and calculated a diabetes severity index based on an adapted Diabetes Complication Severity Index (DSI) [42, 58]. The DSI aims to systematically quantify diabetes complications and includes seven complications of diabetes [retinopathy, nephropathy, neuropathy, cerebrovascular, cardiovascular, peripheral vascular disease (PVD), metabolic disease]. Furthermore, we added risk factors according to the United Kingdom Prospective Diabetes Study and the US Centers for Disease Control and Prevention/RTI diabetes model to our regression analysis [58, 59]. These were operationalized using dummy variables for the relevant comorbidities for which a diagnosis was coded in the years 2012/2013. For a complication to be included in the DSI and a risk factor to be considered at least one confirmed outpatient or inpatient ICD diagnosis, OPS code, or reimbursement code to be documented in the observation period. Furthermore, we indicated via the M3 multimorbidity index whether patients had multiple diseases [60]. In addition, we used the number of prescribed medications (operationalized as ATC agents) to control for the overall morbidity of patients. In case hospitalizations occurred in 2012/2013, we also included a variable to control for the health status of patients following prior hospitalizations.

Causes of ACSH can be related to the health care system, to physicians, to patients’ personal or social circumstances, or some combination of these [61]. We, therefore, also controlled for the unemployment rate (2012), the number of hospital beds (2012), and the density of internists and GPs in the district in which the patient lived (2015). Germany divides into 401 administrative districts incorporating different levels of population density and structure. In line with previous results, we hypothesized that unemployment would increase the risk of hospitalization [62]. Informed by prior empirical evidence, we assumed that physician density (i.e., internists and GPs in the case of diabetes) in a region would be negatively associated with ACSH [6, 11]. Furthermore, based on prior research we expected hospital bed availability to be positively associated with ACSH [63].

### Analytical approach

We explored how spending, continuity and process quality in the ambulatory care sector affects ACSH using a negative binomial model with random effects at the district level. As the dependent variable “hospitalizations due to ACSC” showed a right-skewed distribution with a variance exceeding the mean value, we performed the Vuong closeness test to rule out the possibility that a zero-inflated negative binomial model would have been the better choice. Most variables were available at the individual level. However, the unemployment rate and the variables related to health system access (i.e., internist and GP density, hospital density) were aggregated at the level of districts, giving the model two levels of analysis (i.e., individual and district). We controlled for this by means of random effects.

We undertook multiple regression analyses to assess the association between ambulatory care characteristics and health outcomes in an iterative manner. We first analyzed the effect of ambulatory care spending on health outcomes. To address the effects of coordinated care, we also added a continuity of care index and the number of visits to general practitioners and internists to our second regression analysis. Finally, to capture the additional effect of effective care on hospitalizations, we added the variable indicating whether the recommended processes had been conducted to our third regression.

#### Instrumental variable approach

An unbiased estimation of the coefficients of the regression equation requires that the error term does not correlate with the explanatory variables. However, we do not consider this assumption to be valid in our case. The level of ambulatory care spending and the number of hospitalizations for ACSC may be influenced by unobserved third variables (omitted variables). This could be the case with unrecorded morbidity, which was not measurable in our data and may have influenced the level of ambulatory spending and hospitalizations. Also, the distribution of physicians across Germany was not strictly regulated until the early 1990s [28]. Based on the different regional distribution of morbidity, incentives may have existed for physicians to move to areas with high or low morbidity. In this case, the causality between physician density and hospitalizations may be reversed in the regression (i.e., endogeneity).

An instrumental variable approach was used to control for potential endogeneity of the two variables ambulatory care spending and physician supply with ACSH. The main challenge of the instrumental variable approach was to find an instrument which (a) we could use to control for potential endogeneity of ambulatory care spending and physician density with ACSH but (b) after controlling for exogenous covariates, clearly correlated with the explanatory variable but not with the error term [64]. We chose the approximated average ambulatory care spending per person in a district as this instrument for the individual ambulatory spending. Associations of statutory health insurance physicians negotiate a regional budget for the ambulatory sector with the statutory health insurers. Once physicians exceed the regional budget, they continue to be reimbursed for their services but at progressively lower prices. We thus expected regional average ambulatory care spending to correlate with ambulatory care spending per patient and not with the individual hospitalizations and, therefore, used the former as an instrument for the latter. With regard to physician density in a region, the assumption seems to be met for the number of medical students in a region (2013). We assumed that physicians cluster in proximity to the university at which they are trained [65] and used the number of medical students in a region as an instrument for the physician density in a region.

We undertook a Shorrocks–Shapley decomposition to investigate the relative contribution of groups of regressors to the pseudo *R*^2^ [66]. Here, we aimed to show the total impact of the ambulatory care characteristics under investigation, compared with patients’ morbidity and health care provision variables.

We conducted all analyses using Stata 14.

## Results

### Descriptive statistics

A total of 55,924 patients with type 2 diabetes were included in our analysis (summary statistics are provided in Table 1). The mean age of the patients was 66 years and 50.95% were men. Statutory health insurers spent an average of €820 on ambulatory care per patient each year. In total, 4.80% of the patients received all recommended processes during the observation period. Highest rates are achieved by yearly hbA1c tests (average 87%). Lowest rates are observed with the yearly microalbumin test (average 21%). Patients had a mean continuity of care index of 0.71 and made a mean number of 15 visits to ambulatory care physicians per year.

Overall, 9.82% of the included patients were hospitalized at least once due to an ACSC in 2014. Of these hospitalizations, 14.44% were coded with diabetes as the main diagnosis. Table 2 shows the distribution of hospital cases among the 22 ACSC per 1000 type 2 diabetes patients. Among these conditions, ischemic heart disease was the ACSC with most ACSH followed by diabetes and heart failure. The hospitalization rates are 2.1 times higher than in the general German population with highest differences in ischemic heart diseases, diabetes and heart failure.

**Table 2:**
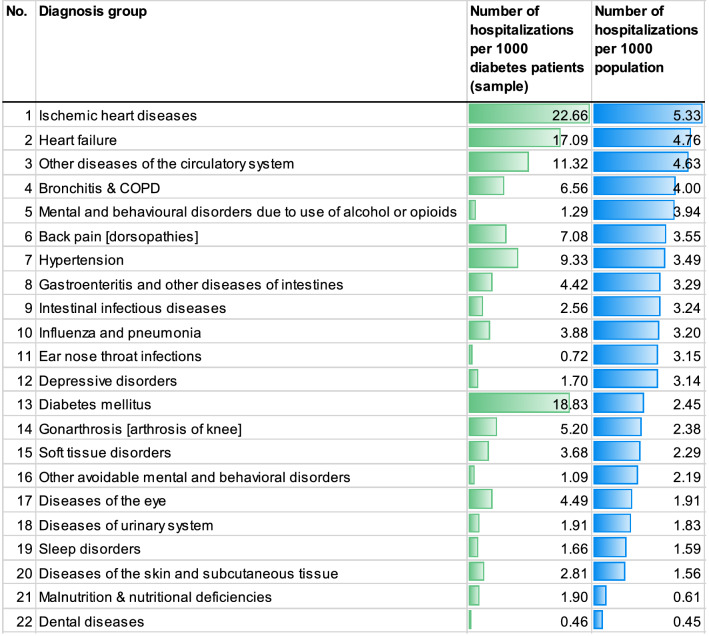
Distribution of hospitalizations among the 22 ACSH in our study compared with Sundmacher et al. [43]

### Regression results

The results of the negative binomial regressions are shown in Table 3. Results of the regressions with instrumental variables are preferred over regular regressions. In the first stage, both instruments exceed the critical value of *F* > 10 established by Staiger and Stock [67] and are thus regarded as valid. The Hausman specification tests also indicate potential endogeneity in all regressions. The following interpretations refer to the results of the instrumental variable regressions.

**Table 3:** Results of the negative binomial model considering random effects

Ambulatory care sensitive hospitalizations	Negative binomial regression						Negative binomial regression with IV					
	IRR	SE	IRR	SE	IRR	SE	IRR	SE	IRR	SE	IRR	SE
**Ambulatory care characteristics**												
Ambulatory care spending (AS)	0.99998	− 0.00001	0.99997*	− 0.00001	0.99997*	− 0.00001	0.99957	− 0.00026	0.99958	− 0.00026	0.99959	− 0.00026
COCI			0.97688	− 0.05233	0.9768	− 0.05233			0.82928 +	− 0.08607	0.82987 +	− 0.08594
Visits			1.00209**	− 0.00073	1.00211**	− 0.00074			1.00624 +	− 0.00346	1.00592 +	− 0.00341
Process quality					0.97999	− 0.05331					1.22706*	− 0.1080
**Patient morbidity**												
Gender^a^	0.9213**	0.02392	0.92254**	− 0.02396	0.9225**	− 0.02396	0.94469*	− 0.02559	0.94685*	− 0.02571	0.94705*	− 0.02574
Age group^b^ 40–59 years	1.26283	− 0.1929	1.26102	− 0.1927	1.26154	− 0.1928	1.16668	− 0.1809	1.18538	− 0.1829	1.18274	− 0.1827
Age group 60–79 years	1.40832*	− 0.2147	1.40802*	− 0.2149	1.40909*	− 0.2151	1.36298*	− 0.2146	1.40224*	− 0.2172	1.39368*	− 0.2161
Age group > 79 years	1.37496*	− 0.2183	1.37573*	− 0.2187	1.37677*	− 0.2189	1.30097	− 0.2449	1.35703 +	− 0.2431	1.35243 +	− 0.2422
DSI	1.09065***	− 0.00700	1.08916***	− 0.00701	1.08917***	− 0.00701	1.11388***	− 0.01046	1.10828***	− 0.0092	1.10789***	− 0.00914
Insulin prescription	1.48760***	− 0.04002	1.47532***	− 0.04115	1.47591***	− 0.04120	1.61176***	− 0.05950	1.55093***	− 0.05297	1.54426***	− 0.05186
Number of ATC agents	1.44032***	− 0.03465	1.43207***	− 0.03457	1.43077***	− 0.03457	1.55198***	− 0.1615	1.49691***	− 0.1291	1.49455***	− 0.1284
Multimorbidity	1.00721	− 0.00498	1.00565	− 0.00500	1.00567	− 0.00501	1.05257*	− 0.02724	1.04271*	− 0.02197	1.04199*	− 0.02179
Prior hospitalizations	1.80455***	− 0.01031	1.80452***	− 0.01032	1.80432***	− 0.01033	1.77010***	− 0.02755	1.76531***	− 0.02553	1.76692***	− 0.02526
Hypertension	1.08228	− 0.07408	1.07986	− 0.07395	1.0799	− 0.07395	1.11779	0.09084	1.11953	− 0.09043	1.11948	− 0.09033
Hazardous alcohol consumption/smoking	1.02701	− 0.0342	1.0265	− 0.03420	1.02624	− 0.03420	0.99456	− 0.03476	0.99213	− 0.03514	0.99522	− 0.0350
Depression	1.03436	− 0.02950	1.03334	− 0.02948	1.03309	− 0.02948	1.12486*	− 0.04669	1.11699**	− 0.04424	1.11762**	− 0.0445
Obesity	1.10832***	− 0.02957	1.11098***	− 0.02964	1.10983***	− 0.02971	1.07409*	− 0.03119	1.07213*	− 0.03183	1.07284*	− 0.0317
Cancer	0.91920*	− 0.03050	0.92407*	− 0.03069	0.92400*	− 0.03069	1.03029	− 0.08478	1.02917	− 0.08305	1.02733	− 0.0825
Sleeping disorder	1.02761	− 0.02999	1.02566	− 0.02994	1.02563	− 0.02994	1.04267	− 0.04307	1.03492	− 0.04023	1.03430	− 0.04013
**Health care provision**												
Physician density	0.95481 +	− 0.02491	0.95474**	− 0.02496	0.95500*	− 0.02498	0.16831**	− 0.1112	0.16964**	− 0.1123	0.16893**	− 0.1118
Hospital bed density	0.99549	− 0.00076	0.99591	− 0.00077	0.99589	− 0.00077	1.26741**	− 0.1154	1.26634**	− 0.1155	1.26684**	− 0.1154
Unemployment rate	1.00382	− 0.00745	1.00342	0.007476	1.00340	− 0.00746	0.90134**	− 0.03619	0.90259*	− 0.03632	0.90260*	− 0.03624
Constant	0.73232	0.32371	0.72520	− 0.32024	0.72299	− 0.31897	2879.204*	8915.831	2905.33**	− 9044.50	2970.533*	− 9248.06
*Observations*	55,924		55,924		55,924		55,924		55,924		55,924	
*Log likelihood*	− 16.906.5		− 16.902.0		− 16.901.9		− 16.903.3		− 16.900.3		− 16.900.1	
*F* *test for AS instrument*							509.22		577.47		581.05	
*F* *test for internists instrument*							82.29		82.27		82.38	

*IRR* incidence rate ratio, *SE* standard error, *AS* ambulatory care spending, *COCI* continuity of care index, *DSI* adapted diabetes complication severity index

^a^Reference group is male

^b^Reference group is aged 18–39 years

Notes: ^+^*p* < 0.10

^*^p < 0.05

^**^p < 0.01

^***^p < 0.001

The first two columns show the results for the effect of ambulatory care spending only. The columns in the middle give the results of the regression models that incorporated the continuity of care index and the number of physician visits. The last two columns show the results of the models that included the measure of effective ambulatory care. All coefficients are reported as incidence rate ratios (IRR) and indicate the factor change in ACSH when an explanatory factor increases by 1 unit, holding all other variables constant. An incidence ratio of 1, for example, suggests that the explanatory variable is a neutral factor in the distribution of hospitalizations between patients. An incidence ratio of 1.5 suggests that an increase in the explanatory variable of 1 unit is associated with a 50% increase in the number of ACSH for the patient.

In all three models, we found a weak negative association between ambulatory care spending and health outcomes, indicating that an increase in ambulatory care spending is associated with fewer ACSH even when controlling for continuous and effective care.

A negative association also existed for continuity of care and hospitalizations. We found that an increase in the continuity of care index from 0 to 1 was associated with a 17% reduction in the number of hospitalizations. The negative association also holds for the alternative measures of continuity of care with GPs only and the wider set of physicians involved in the treatment of diabetes patients and the modified continuity index (see supplementary material). A higher number of physician visits was associated with a higher number of hospitalizations. The variable for effective care was associated with an 23% increase in the number of hospitalizations.

Moreover, when looking at our control variables for morbidity and prior health care use, there was a positive association with the number of hospitalizations resulting from ACSC. Patients with prior hospitalizations are expected to experience 76–77% more hospitalizations. In addition, as expected, hypertension, depression, obesity, and sleeping disorders were positively associated with hospitalizations. A higher number of ATC agents was associated with a higher number of hospitalizations. Women had 5% fewer hospitalizations than men.

Health sector variables showed the expected associations with the number of hospitalizations. Hospital bed density and the number of hospitalizations were positively associated. Physician density was associated with fewer hospitalizations.

The results of the Shapley decomposition analysis for the three groups of variables indicate the following: ambulatory care characteristics (including spending, continuity of care, and the variable for effective care) account for 9.8% of the pseudo *R*^2^, morbidity of patients (including gender and age groups) for about 85.5%, and system-related factors of health provision for 4.7%.

## Discussion

Many unplanned hospital admissions are undesirable for patients and disruptive and costly for health care systems [24]. Those that are caused by ACSC account for a particularly large amount of health spending [68]. A substantial proportion of these hospitalizations can probably be avoided, however, if patients have good access to effective ambulatory care [24–26]. We, therefore, investigated the effect of ambulatory care spending on avoidable hospitalizations in a sample of 55,924 patients with type 2 diabetes.

The risk of hospitalization following ACSC in this group is very high. Compared with the general public in Germany [43], patients in our sample had rates of hospitalization that were four times higher for ischemic heart diseases, three times higher for heart failure, and eight times higher for diabetes. Overall, hospitalization rates resulting from ACSC were 2.1 times higher in our sample than in the general population in Germany, which is similar to differences in rates seen in comparable settings [44].

We find evidence that ambulatory care plays an important role in the management of patients with diabetes, including the prevention of avoidable hospitalizations. In our analysis, which was based on individual-level data from type 2 diabetes patients, we found high variation in costs among patients and a weak negative association between ambulatory care spending and potentially avoidable hospitalizations. After controlling for coordinated and effective care, the relationship between ambulatory care resourcing and hospitalizations remained unchanged. Ambulatory care spending was negatively associated with the number of hospitalizations. While previous literature focused on studying the relationship of resources, such as the physician density, physician visits or operating hours and ACSH [11], our study adds to the previous literature with important information on the effect of health care spending on ACSH.

We find in line with the results of earlier studies that continuity of care has a positive effect on ACSH [26, 69]. Having regular contact with a physician may decrease the likelihood of hospitalization [70]. Continuity of care may facilitate an effective and trusting relationship between patients and physicians and lead to patients understanding their disease better and, therefore, also to greater adherence to treatment or preventive measures [24]. The fragmentation between the ambulatory care and hospital sectors in Germany is a well-known barrier to well-coordinated and continuous care [71].

Furthermore, we did not find that effective care was associated with a lower number of hospitalizations. The coefficient was not significant. Also in earlier studies, the association of diabetes processes of care and hospitalizations in patients with type 2 diabetes was unclear [18]. In absolute terms, we observed that only ~ 5% of our sample received the recommended care indicating regular blood glucose, eye, and microalbumin examination as well as an influenza vaccination. Initiatives to promote adherence to these, where appropriate, may help to reduce hospitalizations and also have other positive effects [19].

In our decomposition analysis, approximately one tenth of the pseudo *R*^2^ could be explained by variables related to access to ambulatory care. An even greater part could be accounted for by variables related to morbidity. In the regression analysis, we found that especially the variables for the number of medications and prior hospitalizations had an impact on the number of hospitalizations. Patients with a higher number of medications resulting from a larger number of comorbidities and prior hospitalizations are those who are most sensitive to discontinuities in care [44, 72]. Policy makers designing initiatives to promote effective and continuous care may want to target these risk groups if a broader population-based approach is not desired or possible.

Our study has several important limitations. First, our sample was drawn from routine data from one major group of statutory health insurers in Germany, which insures approximately one third of all individuals with statutory health insurance in Germany. Our results may not be generalizable to the overall population [73]. Second, our data did not allow us to capture fully the health status of patients. We strove to include a homogeneous patient population by excluding certain conditions, and by controlling for disease severity, risk factors, and comorbidity. However, we cannot rule out that patients’ disease progression was not adequately depicted. Third, we could not include patient preferences or socioeconomic variables at a patient level in our analyses even though these have been shown to be highly relevant to the utilization of health care [61, 62]. We did, however, include socioeconomic status at a regional level based on the assumption that this would approximate patient-level differences.

Furthermore, ambulatory care spending does not always correlate with the number of visits to physicians, because ambulatory care spending is driven mainly by quarterly capitation-type lump sum payments and only specific services are billed additionally. The number of visits to physicians and continuity of care are thus subject to these limitations in routine data.

Last, we attempted to describe quality of care using a composite process indicator. No indicator, however, perfectly describes the quality of care, and individual medical conditions and patient preferences may justify deviations from the recommendations made in clinical practice guidelines [74]. We cannot rule out that our process indicator captured decisions driven by patient preferences and behavior. We aimed to include indicators with low medical uncertainty to depict the effects of effective care rather than the results of preferences for care. However, there remains some uncertainty on the effectiveness of procedures for individual patients. Finally, the results of effective ambulatory care may require more than 2 years to become apparent.

In summary, we provide weak evidence that increased spending and improved continuity of care in the ambulatory care sector may reduce hospitalizations and should be considered when promoting ambulatory care initiatives for people living with chronic illnesses.

## Supplementary Information

Below is the link to the electronic supplementary material.

Supplementary file1 (PDF 286 kb)

## Data Availability

Data provided by the insurance fund cannot be provided due to data protection regulations.

## Code availability

Upon request.
