# Supplementary material for: The impact of ambulatory care spending, continuity and processes of care on ambulatory care sensitive hospitalizations
Source: Eur J Health Econ. 2022 Jan 29;23(8):1329–40. doi: 10.1007/s10198-022-01428-y (PMC9550748; doi:10.1007/s10198-022-01428-y)
Supplement: Supplementary file 1 — Supplementary file1 (PDF 286 kb) [file 10198_2022_1428_MOESM1_ESM.pdf]

Appendix

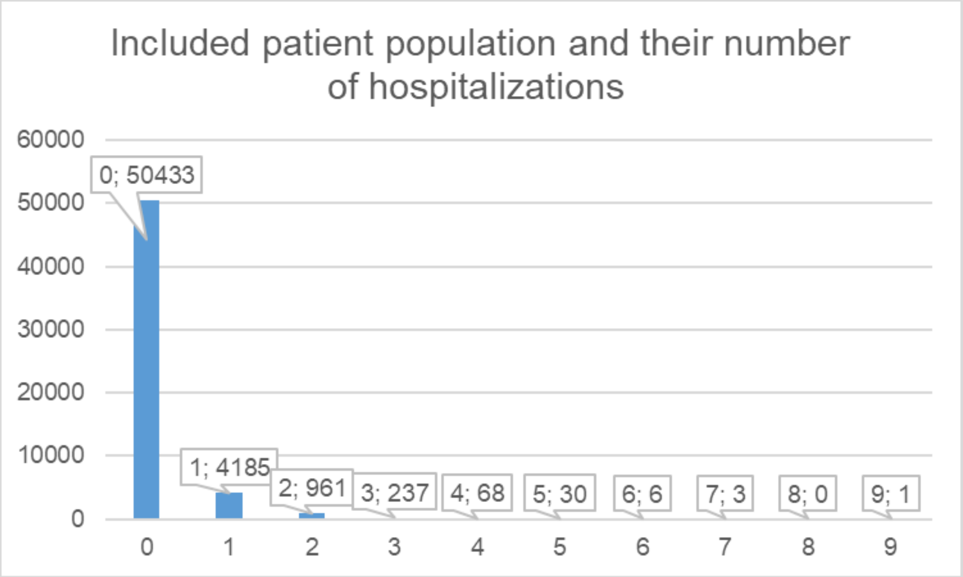

Figure A1: Included patient population and their number of hospitalizations

The **modified modified continuity index** focuses on the dispersion between providers and is only based on the number of providers and the number of visits.

Modified Modified Continuity Index (MMCI) = $\frac{1-\frac{k}{N+0.1}}{1-\frac{1}{N+0.1}}$

k = number of providers

N = total number of visits to all providers

Box A1: Continuity of care index: modified modified continuity index

The DSI war calculated on ICD codes of Kähm et al. 2018; von Ferber, Köster, and Hauner 2007.

**Complication**	**Score**
Retinopathy	0-2
Nephropathy	0-2
Neuropathy	0-1
Cerebrovascular	0-2
Cardiovascular	0-2
Peripheral vascular disease (PVD)	0-2
Metabolic	0-2

Scores per complication rage from 0-2, the sum of complication indicated as DSI thus ranges from 0-13.

Box A2: Components of the adapted diabetes complication severity index (DSI) according to Kähm et al. 2018 and Ferber, Köster and Hauner 2007

Ambulatory care sensitive hospitalizations	Negative binomial regression						Negative binomial regression with IV					
	IRR	SE	IRR	SE	IRR	SE	IRR	SE	IRR	SE	IRR	SE
Ambulatory care characteristics												
Ambulatory care spending (AS)	0.99998	-0.00001	0.99997 *	-0.00001	0.99997 *	-0.00001	0.99921 ***	-0.00024	0.99922 **	-0.00024	0.99922 **	-0.00024
COCI			0.9796	-0.05260	0.97951	-0.05260			0.73232 **	-0.07250	0.73155 **	-0.07250
Visits			1.00195 **	-0.00070	1.00196 **	-0.00071			1.01139 ***	-0.00321	1.01126 ***	-0.00317
Process quality					0.98329	-0.05370					1.13395 +	-0.07910
Patient morbidity												
Gender[1]	0.92981 **	-0.02430	0.93112 **	-0.02430	0.93107 **	-0.02430	0.92639 **	-0.02430	0.93031 **	-0.02450	0.93043 **	-0.02450
Age group[2] 40–59 years	1.30284	-0.20000	1.30234 +	-0.20000	1.30282 +	-0.20000	1.21338	-0.18800	1.2514	-0.19300	1.24924	-0.19200
Age group 60–79 years	1.46303 *	-0.22400	1.46477 *	-0.22400	1.46572 *	-0.22500	1.30157 +	-0.20400	1.37504 *	-0.21300	1.36899 *	-0.21200
Age group >79 years	1.42738 *	-0.22700	1.43045 *	-0.22800	1.43139 *	-0.22800	1.06895	-0.19400	1.15783	-0.20000	1.15351	-0.20000
DSI	1.09255 ***	-0.00699	1.09115 ***	-0.00699	1.09116 ***	-0.00699	1.11188 ***	-0.00997	1.10123 ***	-0.00822	1.10095 ***	-0.00817
Insulin prescription	1.49038 ***	-0.04160	1.47912 ***	-0.04160	1.47963 ***	-0.04170	1.58185 ***	-0.05600	1.47563 ***	-0.04210	1.47161 ***	-0.04190
Number of ATC agents	1.44522 ***	-0.03460	1.43614 ***	-0.03460	1.43618 ***	-0.03460	1.9382 ***	-0.17400	1.81362 ***	-0.13500	1.81423 ***	-0.13500
Multimorbidity	1.00524	-0.00492	1.00382	-0.00494	1.00383	-0.00494	1.08405 ***	-0.02580	1.06721 **	-0.02140	1.06728 **	-0.02140
Prior hospitalizations	1.79790 ***	-0.01030	1.79792 ***	-0.01030	1.79776 ***	-0.01030	1.82627 ***	-0.01530	1.81611 ***	-0.01330	1.81726 ***	-0.01360
Hypertension	1.07516	-0.07390	1.07298	-0.07380	1.07302	-0.07380	0.96859	-0.07140	0.97382	-0.07130	0.97298	-0.07130
Hazardous alcohol consumption/smoking	1.03905	-0.03460	1.03902	-0.03460	1.03876	-0.03460	1.02863	-0.03680	1.02302	-0.03720	1.0249	-0.03700
Depression	1.04944 +	-0.03000	1.04804	-0.03000	1.04784	-0.03000	1.15282 ***	-0.04390	1.13658 ***	-0.04100	1.13726 ***	-0.04120
Obesity	1.12250 ***	-0.03000	1.12409 ***	-0.03010	1.12417 ***	-0.03010	1.08592 **	-0.03100	1.08021 **	-0.03170	1.08033 **	-0.03170
Cancer	0.82660 ***	-0.02760	0.83115 ***	-0.02780	0.83109 ***	-0.02780	1.05182	-0.08620	1.04769	-0.08550	1.04794	-0.08560
Sleeping disorder	1.01295	-0.02990	1.01122	-0.02980	1.01121	-0.02980	1.06835 *	-0.03590	1.05754 +	-0.03440	1.05746 +	-0.03440
Health care provision												
Internist density	0.99012 ***	-0.00282	0.99023 ***	-0.00283	0.99022 ***	-0.00283	0.97338 *	-0.01200	0.97319 *	-0.01200	0.97314 *	-0.01200
Hospital bed density	1.00032	-0.00078	1.00035	-0.00078	1.00035	-0.00078	1.00338	-0.00190	1.00349 +	-0.00190	1.00349 +	-0.00190
Unemployment rate	1.00568	-0.00782	1.00508	-0.00784	1.00504	-0.00784	0.99975	-0.00793	1.00027	-0.00791	1.00038	-0.00790
Constant	0.75382 +	-0.27924	0.44779 +	-0.14782	0.44721 +	-0.14753	0.45162	-0.14833	0.81612	-0.30985	0.81971	-0.31111
Observations	56,881		56,881		56,881		56,881		56,881		56,881	
Log likelihood	–17,056.4		–17,052.2		–17,052.2		–17,053.9		–17,051.4		–17,051.3	
F test for AS instrument (F-value)							247.91		291.94		293.4	
F test for internists instrument (F-value)							3271.91		3273.49		3273.69	

Notes: $^{+}$ $p < 0.10$, * $p < 0.05$, ** $p < 0.01$, *** $p < 0.001$; IRR: incidence rate ratio; SE: standard error; [1] Reference group if male; [2] Reference group is aged 18–39 years; AS: ambulatory care spending; COCI: continuity of care index; DSI: adapted diabetes complication severity index

Table A1: Results of the negative binomial model considering random effects including high-risk patients and patients with HIV, cancer, or receiving opioids

Ambulatory care sensitive hospitalizations	Negative binomial regression				Negative binomial regression with IV			
	IRR	SE	IRR	SE	IRR	SE	IRR	SE
Ambulatory care characteristics								
Ambulatory care spending (AS)	0.99998 +	-0.00001	0.99998 +	-0.00001	0.99926 **	-0.00024	0.99926 **	-0.00024
COCI GP	0.98097	-0.05230	0.98083	-0.05230	0.77580 **	-0.06560	0.77516 **	-0.06550
Visits GP	1.00168 *	-0.00075	1.00170 *	-0.00075	1.00990 ***	-0.00300	1.00977 ***	-0.00295
Process quality			0.97726	-0.05320			1.11995	-0.07840
Patient morbidity								
Gender[1]	0.92089 **	-0.02390	0.92082 **	-0.02390	0.92298 **	-0.02430	0.92310 **	-0.02430
Age group[2] 40–59 years	1.25943	-0.19300	1.26002	-0.19300	1.19905	-0.18400	1.19738	-0.18400
Age group 60–79 years	1.40448 *	-0.21500	1.40570 *	-0.21500	1.30636 +	-0.20200	1.30137 +	-0.20200
Age group >79 years	1.37159 *	-0.21800	1.37279 *	-0.21800	1.10812	-0.19300	1.10467	-0.19300
DSI	1.08942 ***	-0.00702	1.08943 ***	-0.00702	1.09951 ***	-0.00836	1.09932 ***	-0.00832
Insulin prescription	1.47902 ***	-0.04120	1.47971 ***	-0.04130	1.47753 ***	-0.04190	1.47377 ***	-0.04170
Number of ATC agents	1.43404 ***	-0.03470	1.43410 ***	-0.03470	1.79656 ***	-0.13600	1.79753 ***	-0.13600
Multimorbidity	1.00581	-0.00501	1.00583	-0.00501	1.06850 **	-0.02230	1.06844 **	-0.02220
Prior hospitalizations	1.80334 ***	-0.01030	1.80310 ***	-0.01030	1.82680 ***	-0.01510	1.82793 ***	-0.01540
Hypertension	1.07747	-0.07390	1.07748	-0.07390	0.98262	-0.07170	0.98199	-0.07170
Hazardous alcohol consumption/smoking	1.02859	-0.03430	1.02825	-0.03430	1.01377	-0.03690	1.01540	-0.03670
Depression	1.03498	-0.02960	1.03470	-0.02960	1.12176 **	-0.04120	1.12247 **	-0.04140
Obesity	1.11071 ***	-0.02970	1.11079 ***	-0.02970	1.07102 *	-0.03120	1.07128 *	-0.03120
Cancer	0.92279 *	-0.03070	0.92269 *	-0.03070	1.13015	-0.08660	1.13037	-0.08660
Sleeping disorder	1.02610	-0.03000	1.02606	-0.03000	1.09405 *	-0.03940	1.09411 *	-0.03940
Health care provision								
Internist density	0.99081 **	-0.00285	0.99080 **	-0.00285	0.97291 *	-0.01210	0.97286 *	-0.01210
Hospital bed density	1.00023	-0.00079	1.00024	-0.00079	1.00344 +	-0.00192	1.00344 +	-0.00192
Unemployment rate	1.00473	-0.00790	1.00469	-0.00790	0.99966	-0.00799	0.99976	-0.00798
Constant	0.61509	-0.24506	0.61371	-0.24418	1.15467	-0.51104	1.15790	-0.51171
Observations	55,924		55,924		55,924		55,924	
Log likelihood	–16,900.2		–16,900.1		–16,898.1		–16,898.0	
F test for AS instrument					324.48		326.3	
F test for internists instrument					3149.37		3149.54	

Notes: + $p < 0.10$, * $p < 0.05$, ** $p < 0.01$, *** $p < 0.001$; IRR: incidence rate ratio; SE: standard error; [1] Reference group if male; [2] Reference group is aged 18–39 years; AS: ambulatory care spending; COCI: continuity of care index; DSI: adapted diabetes complication severity index

Table A2: Results of the negative binomial model considering random effects with continuity of care based on GPs only

Ambulatory care sensitive hospitalizations	Negative binomial regression						Negative binomial regression with IV					
	IRR		SE	IRR		SE	IRR		SE	IRR		SE
Ambulatory care characteristics												
Ambulatory care spending (AS)	0.99997	*	-0.00001	0.99997	*	-0.00001	0.99923	**	-0.00025	0.99923	**	-0.00025
COCI wide	0.95664		-0.05610	0.95618		-0.05610	0.55592	**	-0.10200	0.55579	**	-0.10100
Visits wide	1.00213	**	-0.00071	1.00216	**	-0.00071	1.01411	***	-0.00427	1.01407	***	-0.00423
Process quality				0.97300		-0.05300				1.05420		-0.06350
Patient morbidity												
Gender[1]	0.92156	**	-0.02400	0.92148	**	-0.02400	0.91959	**	-0.02410	0.91964	**	-0.02410
Age group[2] 40–59 years	1.25803		-0.19200	1.25872		-0.19300	1.21819		-0.18700	1.21735		-0.18700
Age group 60–79 years	1.39818	*	-0.21300	1.39958	*	-0.21400	1.31118	+	-0.20200	1.30860	+	-0.20200
Age group >79 years	1.36567	*	-0.21700	1.36703	*	-0.21700	1.1223		-0.19300	1.12034		-0.19300
DSI	1.08822	***	-0.00704	1.08822	***	-0.00704	1.08931	***	-0.00723	1.08922	***	-0.00722
Insulin prescription	1.47130	***	-0.04120	1.47204	***	-0.04120	1.40175	***	-0.04350	1.40018	***	-0.04380
Number of ATC agents	1.43138	***	-0.03460	1.43144	***	-0.03460	1.74095	***	-0.11600	1.74217	***	-0.11600
Multimorbidity	1.00545		-0.00501	1.00547		-0.00501	1.05839	**	-0.01910	1.05848	**	-0.01910
Prior hospitalizations	1.80439	***	-0.01030	1.80412	***	-0.01040	1.82778	***	-0.01510	1.82834	***	-0.01530
Hypertension	1.07891		-0.07400	1.07896		-0.07400	0.99453		-0.07140	0.99404		-0.07140
Hazardous alcohol consumption/smoking	1.03070		-0.03440	1.03032		-0.03440	1.03258		-0.03620	1.03328		-0.03610
Depression	1.03541		-0.02960	1.03508		-0.02960	1.13376	**	-0.04410	1.13406	**	-0.04420
Obesity	1.11138	***	-0.02970	1.11148	***	-0.02970	1.07552	*	-0.03090	1.07568	*	-0.03090
Cancer	0.92441	*	-0.03070	0.92430	*	-0.03070	1.13303		-0.08780	1.13339	*	-0.08770
Sleeping disorder	1.02669		-0.03000	1.02666		-0.03000	1.09838	*	-0.04020	1.09847		-0.04020
Health care provision												
Internist density	0.99089	**	-0.00285	0.99088	**	-0.00285	0.97307	*	-0.0121	0.97302	*	-0.01210
Hospital bed density	1.00023		-0.00079	1.00024		-0.00079	1.00341	+	-0.00191	1.00342	+	-0.00191
Unemployment rate	1.00448		-0.00790	1.00443		-0.00790	1.00047		-0.00794	1.00049		-0.00793
Constant	0.62633		-0.24976	0.62456		-0.24853	1.37098		-0.61712	1.37130		-0.61631
Observations	55,924			55,924			55,924			55,924		
Log likelihood	–16,898.0			–16,897.9			–16,897.2			–16,897.0		
F test for AS instrument							332.75			333.9		
F test for internists instrument							3153.94			3154.06		

Notes: $^{+}$ $p < 0.10$, * $p < 0.05$, ** $p < 0.01$, *** $p < 0.001$; IRR: incidence rate ratio; SE: standard error; [1] Reference group if male; [2] Reference group is aged 18–39 years; AS: ambulatory care spending; COCI: continuity of care index; DSI: adapted diabetes complication severity index

Table A3: Results of the negative binomial model considering random effects with continuity of care based on GP, internists, eye specialist, diabetic specialist, specialist for nephrology

Ambulatory care sensitive hospitalizations	Negative binomial regression						Negative binomial regression with IV					
	IRR		SE	IRR		SE	IRR		SE	IRR		SE
Ambulatory care characteristics												
Ambulatory care	0.99997	*	-0.00001	0.99997	*	-0.00001	0.99926	**	-0.00024	0.99926	**	-0.00024
MMCOCI	0.79164		-0.15400	0.79240		-0.15500	0.33154	***	-0.10600	0.32908	***	-0.10500
Visits	1.00229	**	-0.00074	1.00231	**	-0.00074	1.01303	***	-0.00375	1.01292	***	-0.00370
Process quality				0.97629		-0.05320				1.11413		-0.07680
Patient morbidity												
Gender[1]	0.92210	**	-0.02400	0.92204	**	-0.02400	0.92712	**	-0.02450	0.92723	**	-0.02450
Age group[2] 40–59 years	1.26798		-0.19400	1.26854		-0.19400	1.21827		-0.18700	1.21677		-0.18700
Age group 60–79 years	1.41548	*	-0.21600	1.41668	*	-0.21700	1.33056	+	-0.20500	1.32586	+	-0.20500
Age group >79 years	1.38393	*	-0.22000	1.38509	*	-0.22100	1.13347		-0.19600	1.13017		-0.19600
DSI	1.08898	***	-0.00702	1.08899	***	-0.00702	1.09749	***	-0.00808	1.09731	***	-0.00805
Insulin prescription	1.47562	***	-0.04100	1.47634	***	-0.04100	1.47253	***	-0.04130	1.46905	***	-0.04120
Number of ATC agents	1.43042	***	-0.03460	1.43050	***	-0.03460	1.77501	***	-0.12900	1.77608	***	-0.12900
Multimorbidity	1.00515		-0.00502	1.00517		-0.00502	1.06448	**	-0.02110	1.06446	**	-0.02100
Prior hospitalizations	1.80344	***	-0.01030	1.80320	***	-0.01030	1.82335	***	-0.01430	1.82442	***	-0.01460
Hypertension	1.07888		-0.07400	1.07888		-0.07400	0.98474		-0.07170	0.98409		-0.07170
Hazardous alcohol consumption/smoking	1.02750		-0.03430	1.02715		-0.03430	1.01221		-0.03690	1.01371		-0.03680
Depression	1.03397		-0.02950	1.03368		-0.02950	1.12001	**	-0.04090	1.12069	**	-0.04110
Obesity	1.11125	***	-0.02970	1.11134	***	-0.02970	1.07366	*	-0.03100	1.07390	*	-0.03100
Cancer	0.92511	*	-0.03080	0.92501	*	-0.03080	1.13171		-0.08620	1.13203		-0.08620
Sleeping disorder	1.02567			1.02563			1.09199	*		1.09205	*	
Health care provision												
Internist density	0.99083	**	-0.00285	0.99082	**	-0.00285	0.97299	*	-0.01210	0.97294	*	-0.01210
Hospital bed density	1.00023		-0.00079	1.00024		-0.00079	1.00344	+	-0.00192	1.00344	+	-0.00192
Unemployment rate	1.00485		-0.00791	1.00480		-0.00791	0.99973		-0.00800	0.99983		-0.00799
Constant	0.74769		-0.32609	0.74475		-0.32427	2.52413	+	-1.33888	2.54592	+	-1.35221
Observations	55924			55924			55924			55924		
Log likelihood	–16,898.1			–16,897.5			–16,898.0			–16,896.4		
F test for AS instrument							319.65			321.39		
F test for internists instrument							3151.99			3152.13		

Notes: $^{+}$ $p < 0.10$, * $p < 0.05$, ** $p < 0.01$, *** $p < 0.001$; IRR: incidence rate ratio; SE: standard error; [1] Reference group if male; [2] Reference group is aged 18–39 years; AS: ambulatory care spending; COCI: continuity of care index; DSI: adapted diabetes complication severity index

Table A4: Results of the negative binomial model considering random effects with the modified modified continuity index
